# Recurrence of postpartum hemorrhage, maternal and paternal contribution, and the effect of offspring birthweight and sex: a population-based cohort study

**DOI:** 10.1007/s00404-021-06374-3

**Published:** 2022-01-09

**Authors:** Lorentz Erland Linde, Cathrine Ebbing, Dag Moster, Jörg Kessler, Elham Baghestan, Mika Gissler, Svein Rasmussen

**Affiliations:** 1 Department of Clinical Science, University of Bergen, Bergen, Norway; 2 Department of Obstetrics and Gynaecology, Haukeland University Hospital, Bergen, Norway; 3 Department of Global Public Health and Primary Care, University of Bergen, Bergen, Norway; 4 Department of Pediatrics, Haukeland University Hospital, Bergen, Norway; 5 Department of Information Services, Finnish Institute for Health and Welfare, Helsinki, Finland; 6 Department of Molecular Medicine and Surgery, Karolinska Institute, Stockholm, Sweden

**Keywords:** Adjusted population attributable fraction, Birthweight, Fetal sex, Inter-delivery interval, Paternal contribution, Postpartum hemorrhage

## Abstract

**Purpose:**

This study examines individual aggregation of postpartum hemorrhage (PPH), paternal contribution and how offspring birthweight and sex influence recurrence of PPH. Further, we wanted to estimate the proportion of PPH cases attributable to a history of PPH or current birthweight.

**Methods:**

We studied all singleton births in Norway from 1967 to 2017 using data from Norwegian medical and administrational registries. Subsequent births in the parents were linked. Multilevel logistic regression was used to calculate odds ratios (ORs) with 95% confidence intervals (CI) for PPH defined as blood loss > 500 ml, blood loss > 1500 ml, or the need for blood transfusion in parous women. Main exposures were previous PPH, high birthweight, and fetal sex. We calculated adjusted population attributable fractions for previous PPH and current high birthweight.

**Results:**

Mothers with a history of PPH had three- and sixfold higher risks of PPH in their second and third deliveries, respectively (adjusted OR 2.9; 95% CI 2.9–3.0 and 6.0; 5.5–6.6). Severe PPH (> 1500 ml) had the highest risk of recurrence. The paternal contribution to recurrence of PPH in deliveries with two different mothers was weak, but significant. If the neonate was male, the risk of PPH was reduced. A history of PPH or birthweight ≥ 4000 g each accounted for 15% of the total number of PPH cases.

**Conclusion:**

A history of PPH and current birthweight exerted strong effects at both the individual and population levels. Recurrence risk was highest for severe PPH. Occurrence and recurrence were lower in male fetuses, and the paternal influence was weak.

**Supplementary Information:**

The online version contains supplementary material available at 10.1007/s00404-021-06374-3.

## Introduction

Postpartum hemorrhage (PPH) is the main direct cause of maternal death worldwide [1], and its incidence is increasing in developed countries [2]. In 2008–2009 PPH occurred in more than 30% of deliveries in UK maternity services [3]. PPH may occur due to uterine atony, genital-tract trauma, placenta-related complications, coagulation disorders or uterine distention caused by a large fetus, multiple pregnancies and polyhydramnios [4, 5]. Augmentation of labor, a previous caesarean section, chronic maternal hypertension and a previous PPH are also identified risk factors [3, 5, 6].

High birthweight is both a strong and a generally increasingly common risk factor for PPH [7, 8], but has not been studied systematically as a modifier in the recurrence of PPH. The paternal influence on PPH, which is mediated through the fetus and the placenta, has been studied, but with inconsistent results [9], and it is unknown if there is an effect of the offspring sex on occurrence and recurrence. Differential likelihoods of having a subsequent delivery after deliveries with and without PPH could potentially influence the recurrence risk estimates. This has not been addressed previously. Further, PPH recurrence has only been studied from an individual rather than a population perspective. Performing such studies requires large, longitudinal datasets.

We used nationwide medical and administrational registries to investigate the maternal and paternal contributions to recurrence risk of PPH and temporal variation in recurrence. We assessed the likelihood of having a subsequent delivery after PPH and studied how recurrence is influenced by birthweight and offspring sex. Further, we estimated the proportions of PPH cases in parous women that are attributable to a history of PPH and high birthweight in the current delivery.

## Materials and methods

### Data sources

The Medical Birth Registry of Norway was established in 1967, since when it has been mandatory to register information of all births in Norway [10]. In 1999, a revised version of the notification form was implemented with new variables, including data on maternal smoking. We included singleton pregnancies with a gestational age at birth of ≥ 22 weeks. Gestational age was estimated from the last menstrual period and was based on ultrasonography when data for the last menstrual period were lacking. First, we analyzed deliveries with spontaneous onset or the induction of labor. We then performed analyses with two different selections: (1) including all deliveries and (2) excluding caesarean deliveries. Information on the parental education level and country of birth were provided by Statistics Norway and linked with the birth registry using the unique national identification number of each birth.

### Record linkage

From 1967 to 2017, 3,003,025 births were registered. We linked subsequent births in the parents. To assess the recurrence risk of PPH in a mother, we linked her first and succeeding births in the registry (to a maximum of three births for each mother). Those who had their first birth in 1967 or later were included. The same dataset was used to explore subsequent delivery rate in women with and without a history of PPH. To assess the recurrence risk of PPH through a man who fathered children with different women, we linked birth records of his first and second child. When analyzing the effect of birthweight on recurrence, we identified pairs of first and second, second and third, and third and fourth births in the same mother, which totaled 1 479 584 pairs of births.

### Outcome variables

The main outcome variable was PPH defined as the loss of more than 500 ml of blood during labor or within 24 h postpartum (hereafter referred to as PPH). From 1999, PPH of more than 1500 ml or the need for blood transfusion (regardless of bleeding volume) were additionally recorded (severe PPH). PPH was notified on forms in free text prior to 1999, and thereafter using check boxes [10].

### Independent variables

The main independent variables were a history of PPH in a previous delivery and the birthweight in the current delivery. To assess temporal changes in the occurrence of PPH, we divided the population into birth-year periods. Further, we investigated whether the occurrence and recurrence of PPH were influenced by maternal conditions such as pregestational and gestational diabetes mellitus, chronic hypertension, preeclampsia, operative vaginal delivery (forceps or vacuum), shoulder dystocia and uterine atony. The possible effect of offspring sex on the recurrence risk of PPH was also explored.

These analyses included the following possible confounding factors: maternal age (in 5-year categories), parity, inter-delivery interval, marital status, mother’s country of birth (Norway or eight WHO regions) [11] and level of education (available until 2013). When analyzing recurrence, the period of birth was divided into five groups with approximately equal durations (1967–1977, 1978–1987, 1988–1997, 1998–2007 and 2008–2017). Supporting information (Statistical analysis) includes additional details.

### Statistical analysis

We used multilevel logistic regression analyses to calculate odds ratios (ORs) with 95% confidence intervals (CIs) for PPH in the actual birth as the outcome, and a history of previous PPH was the main exposure variable.

Sensitivity analyses were performed to assess the impact of unmeasured confounders on the recurrence of PPH. We estimated the proportion of all cases of PPH in the Norwegian birth population attributable to previous PPH and current high birthweight (≥ 4000 g) [adjusted population attributable fractions (aPAFs)].

To assess likelihoods of a further delivery after PPH, we calculated further pregnancy rate [12], and used Cox proportional hazards regression of time from the first delivery. Supporting information (Statistical analyses) includes additional details.

The statistical analyses were performed using SPSS (version 25) and MLwiN (version 3.05).

## Results

The study population included 2,790,090 singleton deliveries with a gestational age of at least 22 weeks from 1967 to 2017. PPH was registered in 10% of the deliveries (*n* = 277,746), and the rate of caesarean section was 11% (*n* = 295,920) (Supporting information Table S1). There was an increasing trend of the occurrence of PPH during the study period. Increasing occurrences were also observed in pregnancies with high maternal age, maternal medical conditions and pregnancy-related complications (preeclampsia, operative delivery and placental pathology) (Supporting information Table S1).

The risk of PPH for the total population was lower if the newborn was a boy (OR: 0.96, 95% CI 0.96–0.97). These results remained unchanged by adjustments for parity. After adjusting for birthweight this effect was stronger (aOR: 0.89, 95% CI 0.88–0.90).

While several maternal characteristics and conditions were associated with PPH (Supporting information Table S1), the ORs for the recurrence of PPH changed only marginally after adjusting for known possible confounders (Table 1). However, as an exception, the period of birth moderately decreased the effects on recurrence. When we included the assumption of a strong unknown confounder in addition to period of birth in our sensitivity analyses, the ORs of recurrence decreased by less than 5%. Therefore, in the final regression analyses we mainly adjusted for birth year period only.

**Table 1:** Recurrence of postpartum hemorrhage (PPH) (> 500 ml) according to year of delivery and change of father

		Total	PPH (*n*)	%	OR	95% CI		aOR	95% CI	
First delivery (PPH > 500 ml)		Second delivery								
	No	720 761	49 822	6.9	1	Reference		1	Reference	
	Yes	73 929	16 721	22.6	3.94	3.86	4.01	2.92	2.86	2.98
PPH	Year									
No	1967–1983	217 419	8973	4.1	1	Reference		1	Reference	
Yes		9589	1246	13.0	3.47	3.26	3.70	3.48	3.24	3.73
No	1983–1998	234 571	10 888	4.6	1	Reference		1	Reference	
Yes		14 793	1928	13.0	3.08	2.92	3.24	3.07	2.91	3.24
No	1999–2017	268 771	29 961	11.1	1	Reference		1	Reference	
Yes		49 547	13 547	27.3	3.00	2.93	3.07	2.94	2.87	3.01
PPH	Change of father^a^									
No	No	645 586	44 012	6.8	1	Reference		1	Reference	
Yes		67 547	15 401	22.8	4.04	3.96	4.12	2.98	2.92	3.05
No	Yes	64 097	4914	7.7	1	Reference		1	Reference	
Yes		5274	1059	20.1	3.03	2.81	3.26	2.49	2.31	2.69
First and second deliveries (PPH > 500 ml)		Third delivery								
First	Second									
No	No	247 823	13 876	5.6	1	Reference		1	Reference	
	Yes	14 666	2755	18.8	3.90	3.73	4.08	3.31	3.15	3.47
Yes	No	17 446	2382	13.7	2.67	2.55	2.79	2.10	2.00	2.20
	Yes	3772	1219	32.3	8.05	7.50	8.64	5.62	5.22	6.05

*CI* confidence interval, *OR* odds ratio, *aOR* OR adjusted for period (1967–1977, 1978–1987, 1988–1997, 1998–2007 and 2008–2017)

^a^Additionally adjusted for inter-delivery interval

### Recurrence of bleeding

Mothers with PPH (> 500 ml) in their first delivery had a threefold higher risk of excessive bleeding in their second delivery (Table 1). The probability of recurrence of bleeding decreased significantly during the study period (Table 1). The recurrence risk of PPH was highest if the father was the same in both pregnancies, also after adjustment for the inter-delivery interval. The risk of PPH and recurrent PPH was lower if the newborn was a boy. Stillbirth did not influence the risk of recurrent PPH, but was significantly associated with PPH in women without previous PPH. (Supporting information Table S2). Mothers with three deliveries had the highest recurrence risk of PPH in the third delivery if they had a history of PPH in the two preceding deliveries (Table 1). These effects were slightly stronger when we excluded cesarean deliveries (data not shown). Exclusion of induced deliveries had no effect on recurrence (data not shown). The region of birth of the mother did not affect recurrence (data not shown). From 1999 onwards, when data on severe PPH (> 1500 ml) were available, the risk of severe PPH in the second delivery was higher for mothers with severe PPH in the first delivery (aOR: 6.0, 95% CI 5.5–6.6), than for those with PPH of > 500 ml (aOR: 3.5, 95% CI 3.3–3.7).

Adjusting for factors other than birth year period had negligible effects on the ORs of PPH recurrence (Table 1). However, maternal medical conditions and pregnancy characteristics influenced the occurrence of PPH, but least in women with a history of PPH (Supporting information Table S2). Inter-delivery interval had almost no effect on the risk of PPH in the second birth (Supporting information Table S3).

Tracing men who fathered children with two different women, we found significantly increased risk of recurrent PPH (OR: 1.51, 95% CI 1.40–1.64), including after adjusting for period of birth and inter-delivery interval (aOR: 1.12, 95% CI 1.03–1.21). Adjusting for birthweight had negligible effect.

**Fig. 1:**
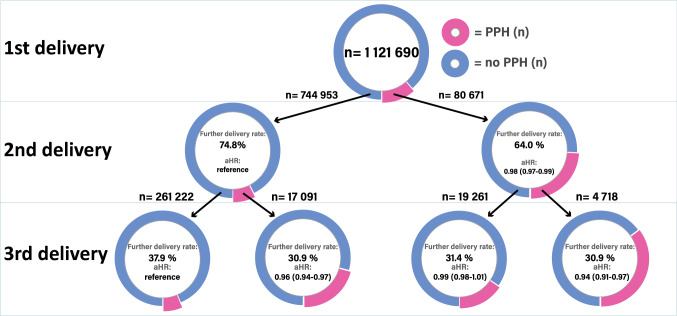
Further delivery rate and adjusted Hazard ratios (aHR) in women with a history of postpartum hemorrhage (PPH) compared to women without

Subsequent delivery rate in the second delivery was lower in mothers who had experienced PPH in the first delivery, compared to those who had not (Fig. 1) (64.0 and 74.8%, respectively). Corresponding adjusted hazard ratios (with 95% CI) confirmed statistically significant differences (unadjusted and adjusted hazard ratio 0.97 (95% CI 0.96–0.97) and 0.98 (0.97–0.99). Exploring subsequent deliveries in women with three deliveries, we found that women with PPH in the first two deliveries had the lowest rate of third deliveries (30.9%), compared with no PPH in both deliveries (37.9%) (unadjusted and adjusted hazard ratios 0.86 (0.84–0.89) and 0.94 (0.91–0.97), respectively) (Fig. 1). The cumulative hazard ratio graphs began to diverge about five and three years after the first (Supporting information Figure S1) and second delivery (Supporting information Figure S2), respectively.

### Combined effect of birthweight in actual pregnancy and PPH anamnesis

We explored the impact of birthweight on the risk of PPH according to the history of PPH (Table 2).

**Table 2:** Impacts of birthweight in the current delivery on the occurrence and recurrence of postpartum hemorrhage (PPH) (> 500 ml)

Recurrence in the same mother										
Birthweight in current delivery	PPH in mother’s previous delivery	PPH in current delivery								
		Total	PPH (*n*)	%	OR	95% CI		aOR^a^	95% CI	
< 4000 g	No	885,286	49,911	5.6	1	Reference		1	Reference	
4000–4499 g	No	206,955	18,705	9.0	1.66	1.63	1.69	1.70	1.67	1.73
4500–4999 g	No	44,314	5667	12.8	2.46	2.38	2.53	2.58	2.50	2.66
≥ 5000 g	No	5762	943	16.4	3.28	3.05	3.52	3.63	3.37	3.90
< 4000 g	Yes	70,363	14,093	20.0	1	Reference		3.01	2.95	3.07
4000–4499 g	Yes	24,833	6471	26.1	1.40	1.36	1.45	4.56	4.42	4.70
4500–4999 g	Yes	6705	2097	31.3	1.81	1.71	1.92	6.24	5.91	6.59
≥ 5000 g	Yes	1026	367	35.8	2.21	1.94	2.53	8.06	7.06	9.21

^a^aOR, OR adjusted for marital status, period (1967–1977, 1978–1987, 1988–1997, 1998–2007 and 2008–2017), maternal age, parity and WHO region of maternal birth

As an example of Table 2, if the mother experienced PPH in her previous delivery and gave birth to a newborn ≥ 5000 g in the current delivery, the risk of PPH was eightfold higher compared with mothers who had no history of PPH giving birth to a newborn weighing less than 4000 g (Table 2).

The results in Table 2 indicate that the birthweight in the current delivery and a history of PPH had additive effects on subsequent PPH risk.

Similarly, the risk of PPH in primiparas (*n* = 1,245,244) was more than fourfold higher (aOR: 4.37, 95% CI 4.00–4.78) when the birthweight was ≥ 5000 g compared with a birthweight of < 4000 g.

### Population attributable fractions

Among all deliveries with PPH in parous women, 14.4% (the value of aPAF, corresponding to 14,166 cases of PPH) was attributable to a history of PPH (with no previous PPH as the reference). Of all deliveries with PPH in parous females, 15.3% (15,015 cases) was attributable to any birthweight above 4000 g in the current delivery (< 4000 g (reference), 4000–4499 g, 4500–4999 g or ≥ 5000 g). Similarly, of all first pregnancies with PPH in the same population, 15.0% (15,486 cases) was attributable to any birthweight above 4000 g.

## Discussion

This study confirmed and quantified that a history of PPH increased the risk of PPH in a mother’s subsequent deliveries. The current birthweight was a strong modifier of recurrent PPH risk. Concomitantly with increasing absolute risks of PPH, the ORs of recurrence decreased slightly by birth year period. We found a weak paternal effect on PPH, and that the risk of PPH was lower if the offspring was a boy. The subsequent delivery rate was lowest in women with a delivery with PPH. A history of PPH and the current birthweight exerted strong effects at both the individual and population levels.

The main strengths of this study were its large size, essentially complete record linkage and the more than 50 years follow-up period, which made it possible to perform comprehensive sub-analyses. The population-based design and prospective collection of data reduced selection and recall biases. The sensitivity analyses indicated that unmeasured confounders did not reduce the reliability of the obtained results. Another strength is that many covariates and possible confounding factors were validated and found to be of adequate quality for utilization in epidemiological studies [13, 14].

When registrations of severe pregnancy complications between 2008 and 2013 were scrutinized, the variable of severe PPH was found to be of acceptable quality, with a sensitivity of 87.7% and a positive predictive value of 81.1% [15]. Keeping in mind that severe PPH is often misclassified as mild PPH [16], we consider the sensitivity of severe PPH in our study to be high.

We cannot rule out that the introduction of activity-based financing of the Norwegian health care system in 1997 and the use of a new notification form in 1999 might have resulted in increased registration (which may imply a higher proportion of false negatives before and/or increased rate of false positives after this introduction). However, it is likely that any such misclassification was non-differential, and thus did not affect the ORs of recurrence. ORs of recurrence decreased slightly during the study period, which was expected since mild PPH was likely to have been underreported during the previous period. Residual confounding caused by unmeasured confounding factors cannot be ruled out, but our sensitivity analyses indicated that this was not present.

The relatively ethnically homogeneous Norwegian birth population might limit the generalizability of our findings to other parts of the world. However, our finding that recurrence rates in immigrants from different regions were similar supports the generalizability of our results. The finding that the risk of PPH in the third delivery followed a dose response pattern to previous births with PPH, and that the recurrence risks were highest in severe PPH, strengthens the biological plausibility of our results.

Our findings for recurrence of PPH are consistent with the results of a Swedish study [17]. Concerning the paternal contribution to PPH, we found that the recurrence risk was significantly increased in deliveries where the father had changed partner which did not reach statistical significance in a Swedish study [9]. This inter-study difference is probably due to the larger sample in our study. However, the higher maternal recurrence risk when the father was the same in both pregnancies is consistent with the Swedish study [9]. Our finding that stillbirth was associated with PPH in women without previous PPH is consistent with earlier studies [18]. However, a new finding was the lack of association in women with previous PPH, which may represent index event bias [19]. However, it cannot be ruled out that stillbirth in women with and without previous PPH have different pathophysiological mechanisms.

Overall, we found the highest recurrence risk of PPH when the study population was restricted to include only vaginal deliveries, which corroborates the findings of the Swedish group [17]. However, we decided to include deliveries with spontaneous onset and induction of labour in order to make the findings more relevant to clinical practice. Because changing practices in induction of labor during the study period potentially influence recurrence risks, we excluded deliveries with induction of labor in a supplementary analysis, but this did not change the risk estimates.

A short inter-pregnancy interval has been associated with adverse perinatal outcomes in some studies [20–22] but not others [23]. We found that the inter-delivery interval had almost no effect on recurrence of PPH (Supporting information Table S3).

While fetal macrosomia has been associated with PPH [24], it has not previously been shown that birthweight influences the recurrence risk (Table 2), which may be explained by mechanisms such as atony caused by uterine distension, and a large uteroplacental wound surface.

Sex differences are present in birthweight, placental weight and umbilical cord properties [25–27], but it was an unexpected finding that delivering female neonates carried a higher risk of PPH, including after adjusting for birthweight. Fetal sex differences in occurrence of PPH have been reported in earlier studies with inconsistent results [28]. However, these studies had methodological weaknesses or were underpowered to answer this question. The finding is difficult to explain, but it is possible that the placentas of female fetuses have different vascular or invasive properties that increase the risk of PPH relative to placental weight and birthweight. One may also speculate if sex-specific preponderance differs between primary causes of PPH such as uterine atony and retained placenta. The effect of fetal sex on PPH risk is interesting from a biological, and possibly evolutionary perspective [29], and generates new research questions into sex differences in the placenta.

Acetylsalicylic acid has been offered to pregnant women at increased risk of developing preeclampsia in Norway since 1999 [30], but is a known risk factor for PPH [31], which may have contributed to the observed increased occurrence of PPH. To explore this further was beyond the scope of this study. During the study period, tranexamic acid to prevent PPH in women at risk was not routinely administered [32].

The recurrence of PPH may be caused by genetic and/or sustained environmental factors. We also found a paternal influence on recurrence, which was weaker than the maternal effect presumably due to paternal genes being limited to the fetus, placenta and decidua (through trophoblast invasion).

Further pregnancy rate after obstetric complications other than PPH has been studied [12]. The lower subsequent delivery rates in women who had experienced PPH were not evident before five and three years after the first and second delivery, respectively (Supporting information Figures S1 and S2). This may be due to a traumatic birth experience associated with PPH and could potentially influence recurrence risk estimates, but the latter is unlikely, since the divergence of the cumulative hazard ratio graphs was delayed.

The present study suggests that the combined history of PPH and anticipated fetal size may be useful in identifying women at risk of PPH. From an individual perspective a history of PPH and birthweight of ≥ 4000 g were the strongest exposure variables, warranting attention to fetal growth and preparedness and attention to exposed mothers during labor. From a public health perspective, a history of PPH and high birthweight in the current delivery have non-negligible impacts on the total number of PPH in the population. Investigating recurrence patterns between relatives and cause-specific PPH (e.g., PPH associated with uterine atony or retained placenta) is warranted.

## Conclusion

This population-based study found that the recurrence risks of PPH was modulated by birthweight and had a modest paternal, and offspring sex influence. These effects were consistent throughout the 50-year study period despite the trend of increasing occurrence. Our findings add to the understanding of recurrence of PPH and may be relevant for health care personnel who are counselling mothers with a history of PPH.

## Supplementary Information

Below is the link to the electronic supplementary material.

Supplementary file1 Table S1: Postpartum hemorrhage (PPH) (>500 ml) according to maternal and pregnancy characteristics of the study population. (DOCX 67 KB)

Supplementary file2 Table S2: Risk of postpartum hemorrhage (PPH) (>500 ml) according to maternal and pregnancy characteristics. (DOCX 169 KB)

Supplementary file3 Table S3: Inter-delivery interval and recurrence risk of postpartum hemorrhage (PPH, >500 ml). (DOCX 52 KB)

Supplementary file4 Figure S1: Cumulative Hazards of the second delivery according to postpartum hemorrhage in the first delivery, adjusted for period and maternal age. (TIF 92 KB)

Supplementary file5 Figure S2: Cumulative Hazards of the third delivery according to postpartum hemorrhage in the first or second delivery, adjusted for period and maternal age. (TIF 123 KB)

Supplementary file6 Statistical analysis. (DOCX 39 KB)

## Data Availability

Legal restrictions do not permit the authors to provide the data that constitute the basis of this study. The main data utilized are available from the data owner, the Norwegian Institute of Public Health (https://www.fhi.no/en/more/research--access-to-data/), after obtaining approval from The Regional Committee for Medical Research Ethics (https://rekportalen.no/), for researchers who meet the criteria for access to confidential data. Contact information: The Medical Birth Registry of Norway, University of Bergen, P.O. Box 7804, 5020 Bergen, Norway.

## Abbreviations

aOR: Adjusted odds ratio
CI: Confidence interval
OR: Odds ratio
PPH: Post-partum hemorrhage

## Code availability

The data are confidential and cannot be shared.
