# Supplementary material for: Recurrence of postpartum hemorrhage, maternal and paternal contribution, and the effect of offspring birthweight and sex: a population-based cohort study
Source: Arch Gynecol Obstet. 2022 Jan 9;306(5):1807–14. doi: 10.1007/s00404-021-06374-3 (PMC9519656; doi:10.1007/s00404-021-06374-3)
Supplement: Supplementary file 1 — Supplementary file1 Table S1: Postpartum hemorrhage (PPH) (>500 ml) according to maternal and pregnancy characteristics of the study population. (DOCX 67 KB) [file 404_2021_6374_MOESM1_ESM.docx]

| **Supplementary Table 1. Postpartum hemorrhage (PPH) (>500 ml) according to maternal and pregnancy characteristics of the study population^a^** | | | |  |
| --- | --- | --- | --- | --- |
|  |  |  |  |  |
| **Characteristic** | **Total (*n*)** | **PPH (*n*)** | **%** |  |
| Total population | **2 790 090** | **277 746** | **10.0** |  |
| **Maternal age (Years)** |  |  |  |  |
| <20 | 137 679 | 8397 | 6.1 |  |
| 20–24 | 664 411 | 49 485 | 7.4 |  |
| 25–29 | 946 744 | 91 753 | 9.7 |  |
| 30–34 | 701 018 | 81 701 | 11.7 |  |
| 35–39 | 284 957 | 38 170 | 13.4 |  |
| 40–44 | 52 693 | 7779 | 14.8 |  |
| 45–49 | 2514 | 443 | 17.6 |  |
| ≥50 | 74 | 18 | 24.3 |  |
| **Parity** |  |  |  |  |
| 0 | 1 161 540 | 135 430 | 11.7 |  |
| 1 | 978 573 | 90 718 | 9.3 |  |
| 2 | 447 693 | 36 037 | 8.0 |  |
| 3 | 135 235 | 10 241 | 7.6 |  |
| ≥4 | 67 049 | 5320 | 7.9 |  |
| **Year of delivery** |  |  |  |  |
| <1970 | 193 336 | 8736 | 4.5 |  |
| 1970–1979 | 553 307 | 22 875 | 4.1 |  |
| 1980–1989 | 481 392 | 22 966 | 4.8 |  |
| 1990–1999 | 535 289 | 35 732 | 6.7 |  |
| 2000–2010 | 561 738 | 84 281 | 15.0 |  |
| >2010 | 465 028 | 103 156 | 22.2 |  |
| **Maternal height^b^** |  |  |  |  |
| <160 cm | 45 610 | 10 745 | 23.6 |  |
| 160–169 cm | 201 739 | 42 374 | 21.0 |  |
| 170–179 cm | 121 785 | 25 442 | 20.9 |  |
| ≥180 cm | 9135 | 2093 | 22.9 |  |
| **WHO region** |  |  |  |  |
| Norway | 2 146 167 | 208 482 | 9.7 |  |
| High-income countries | 96 787 | 11 977 | 12.4 |  |
| Central Europe, Eastern Europe and Central Asia | 61 475 | 11 870 | 19.3 |  |
| Sub-Saharan Africa | 35 276 | 7260 | 20.6 |  |
| North Africa and Middle East | 41 934 | 6323 | 15.1 |  |
| South Asia | 25 942 | 3258 | 12.6 |  |
| Southeast Asia, East Asia and Oceania | 47 532 | 10 360 | 21.8 |  |
| Latin America and Caribbean | 8911 | 1961 | 22.0 |  |
| Unknown or stateless | 410 | 57 | 13.9 |  |
| **Education (years)^c^** |  |  |  |  |
| <8 | 15 284 | 1722 | 11.3 |  |
| 8–10 | 562 009 | 39 809 | 7.1 |  |
| 11–12 | 455 375 | 22 875 | 5.0 |  |
| 13–17 | 1 318 049 | 129 574 | 9.8 |  |
| ≥18 | 162 156 | 20 695 | 12.8 |  |
| Not defined | 40 781 | 5719 | 14.0 |  |
| **Marital status** |  |  |  |  |
| Married/ registered partner | 1 818 200 | 153 432 | 8.4 |  |
| Cohabitating | 713 685 | 102 238 | 14.3 |  |
| Not married/alone | 219 502 | 18 313 | 8.3 |  |
| Divorced / Separated / Widow | 22 467 | 1641 | 7.3 |  |
| Not defined | 16 236 | 2122 | 13.1 |  |
| **Smoking at start of pregnancy^d^** |  |  |  |  |
| No | 797 243 | 145 900 | 18.3 |  |
| Occasionally | 16 011 | 2526 | 15.8 |  |
| Daily | 112 604 | 15 305 | 13.6 |  |
| **Chronic hypertension** | 9446 | 1726 | 18.3 |  |
| **Anemia^e^** | 3068 | 500 | 16.3 |  |
| **Bleeding disorders^f^** | 10 191 | 1976 | 19.4 |  |
| **Pregestational diabetes mellitus** | 11 423 | 2310 | 20.2 |  |
| **Gestational diabetes mellitus** | 25 334 | 6207 | 24.5 |  |
| **Preeclampsia** | 80 321 | 11 886 | 14.8 |  |
| HELLP syndrome^g^ | 1595 | 569 | 35.7 |  |
| **Onset of birth** |  |  |  |  |
| Spontaneous | 2 249 026 | 191690 | 8.5 |  |
| Induction | 429 901 | 58 210 | 13.5 |  |
| Cesarean section | 111 131 | 27 846 | 25.1 |  |
| Not recorded | 32 | 0 | 0.0 |  |
| **Birthweight (grams)** |  |  |  |  |
| <4000 g | 2 262 811 | 199 404 | 8.8 |  |
| 4000–4499 g | 428 460 | 59 709 | 13.9 |  |
| 4500–4999 g | 87 311 | 16 044 | 18.4 |  |
| ≥5000 g | 11 508 | 2589 | 22.5 |  |
| **Newborn’s sex** |  |  |  |  |
| Female | 1 355 794 | 137 251 | 10.1 |  |
| Male | 1 434 080 | 140 477 | 9.8 |  |
| **Mode of delivery** |  |  |  |  |
| Cesarean section | 295 920 | 69 428 | 23.5 |  |
| Vaginal delivery | 2 494 170 | 208 318 | 8.4 |  |
| **Shoulder dystocia** | 20 255 | 3866 | 19.1 |  |
| **Vacuum delivery** | 145 785 | 27 076 | 18.6 |  |
| **Forceps delivery** | 55 766 | 7061 | 12.7 |  |
| **Uterine curettage, retained placenta or placenta accreta** | 96 859 | 35 664 | 36.8 |  |
| **Uterine atony** | 72 484 | 72 484 | 100.0 |  |
| **Genital trauma, hematoma, tear or uterine inversion** | 121 691 | 28 673 | 23.6 |  |
| **Placental abruption** | 14 096 | 3598 | 25.5 |  |
| **Placenta previa** | 6918 | 2542 | 36.7 |  |
| **Dystocia** | 188 234 | 37 597 | 20.0 |  |

Including singleton deliveries, gestational age ≥22 weeks from the last menstrual period or estimated by ultrasonography and specified maternal age, year of birth and birthweight

b Available from 2006–2017

c until 2013

d from 1998 onwards

e ICD-8 codes 280–285; ICD–10 codes D50–D53, D55, D58–D61, D63 and D64

f ICD-8 codes 286–289; ICD–10 codes D56, D57, D62, D65–D77, O460, O670 and O723

g HELLP; Hemolysis, Elevated Liver enzymes, Low Platelets
