# Supplementary material for: Recurrence of postpartum hemorrhage, maternal and paternal contribution, and the effect of offspring birthweight and sex: a population-based cohort study
Source: Arch Gynecol Obstet. 2022 Jan 9;306(5):1807–14. doi: 10.1007/s00404-021-06374-3 (PMC9519656; doi:10.1007/s00404-021-06374-3)
Supplement: Supplementary file 2 — Supplementary file2 Table S2: Risk of postpartum hemorrhage (PPH) (>500 ml) according to maternal and pregnancy characteristics. (DOCX 169 KB) [file 404_2021_6374_MOESM2_ESM.docx]

| **Supplementary Table 2. Risk of postpartum hemorrhage (PPH) (>500 ml) according to maternal and pregnancy characteristics** | | | | | | | | | | | |
| --- | --- | --- | --- | --- | --- | --- | --- | --- | --- | --- | --- |
| **Exposure variable** | | **Outcome: PPH >500 mL in current delivery** | | | | | | | | | |
| **Complication** | **Previous PPH >500 mL** | **Total** | ***n*** | | **%** | OR | 95% CI | | **aOR** | 95% CI | |
| **Anemia^a^** |  |  |  | |  |  |  |  |  |  |  |
| No | No | 1 141 123 | 75 097 | | 6.58 | **1** |  | | **1** |  | |
| Yes | No | 1194 | 129 | | 10.80 | **1.72** | 1.43 | 2.07 | **1.22** | 1.02 | 1.48 |
| No | Yes | 102 737 | 22 962 | | 22.4 | **1** |  | | **1** |  | |
| Yes | Yes | 190 | 66 | | 34.7 | **1.85** | 1.36 | 2.51 | **1.50** | 1.10 | 2.05 |
| **Bleeding disorder^b^** | |  |  | |  |  |  |  |  |  |  |
| No | No | 1 138 416 | 74 788 | | 6.57 | **1** |  | | **1** |  | |
| Yes | No | 3901 | 438 | | 11.23 | **1.80** | 1.63 | 1.99 | **1.07** | 0.97 | 1.18 |
| No | Yes | 102 243 | 22 825 | | 22.3 | **1** |  | | **1** |  | |
| Yes | Yes | 684 | 203 | | 29.7 | **1.46** | 1.24 | 1.73 | **1.11** | 0.94 | 1.32 |
| **Chronic hypertension** | |  |  | |  |  |  |  |  |  |  |
| No | No | 1 138 690 | 74 793 | | 6.57 | **1** |  | | **1** |  | |
| Yes | No | 3627 | 433 | | 11.94 | **1.93** | 1.74 | 2.13 | **1.56** | 1.40 | 1.72 |
| No | Yes | 102 440 | 22 876 | | 22.3 | **1** |  | | **1** |  | |
| Yes | Yes | 487 | 152 | | 31.2 | **1.58** | 1.30 | 1.92 | **1.34** | 1.09 | 1.63 |
| **Pregestational diabetes mellitus** | | |  | |  |  |  |  |  |  |  |
| No | No | 1 138 800 | 74 783 | | 6.6 | **1** |  | | **1** |  | |
| Yes | No | 3517 | 443 | | 12.6 | **2.05** | 1.86 | 2.27 | **1.55** | 1.40 | 1.72 |
| No | Yes | 102 404 | 22 858 | | 22.3 | **1** |  | | **1** |  | |
| Yes | Yes | 523 | 170 | | 32.5 | **1.67** | 1.39 | 2.02 | **1.39** | 1.15 | 1.68 |
| **Gestational diabetes mellitus** | |  |  | |  |  |  |  |  |  |  |
| No | No | 1 133 265 | 73 976 | | 6.5 | **1** |  | | **1** |  | |
| Yes | No | 9052 | 1250 | | 13.8 | **2.29** | 2.16 | 2.44 | **1.28** | 1.20 | 1.36 |
| No | Yes | 100 947 | 22 352 | | 22.1 | **1** |  | | **1** |  | |
| Yes | Yes | 1980 | 676 | | 34.1 | **1.82** | 1.65 | 2.00 | **1.24** | 1.12 | 1.37 |
| **Preeclampsia** |  |  |  | |  |  |  |  |  |  |  |
| No | No | 1 122 874 | 73 324 | | 6.53 | **1** |  | | **1** |  | |
| Yes | No | 19 443 | 1902 | | 9.78 | **1.55** | 1.48 | 1.63 | **1.56** | 1.48 | 1.63 |
| No | Yes | 100 994 | 22 505 | | 22.3 | **1** |  | | **1** |  | |
| Yes | Yes | 1933 | 523 | | 27.1 | **1.30** | 1.17 | 1.44 | **1.29** | 1.16 | 1.44 |
| **Preeclampsia, delivery before 37 weeks** | | |  |  |  |  |  |  |  |  |  |
| No | No | 1 139 693 | 74 947 | | 6.58 | **1** |  | | **1** |  | |
| Yes | No | 2624 | 279 | | 10.63 | **1.69** | 1.49 | 1.91 | **1.66** | 1.46 | 1.88 |
| No | Yes | 102 655 | 22 952 | | 22.4 | **1** |  | | **1** |  | |
| Yes | Yes | 272 | 76 | | 27.9 | **1.35** | 1.03 | 1.77 | **1.24** | 0.94 | 1.63 |
| **Shoulder dystocia** | |  |  | |  |  |  |  |  |  |  |
| No | No | 1 132 619 | 73 985 | | 6.5 | **1** |  | | **1** |  | |
| Yes | No | 9698 | 1241 | | 12.8 | **2.10** | 1.97 | 2.23 | **1.81** | 1.70 | 1.93 |
| No | Yes | 101 637 | 22 583 | | 22.2 | **1** |  | | **1** |  | |
| Yes | Yes | 1290 | 445 | | 34.5 | **1.84** | 1.63 | 2.07 | **1.76** | 1.56 | 1.99 |
| **Vacuum delivery** | |  |  | |  |  |  |  |  |  |  |
| No | No | 1 121 044 | 72 477 | | 6.5 | **1** |  | | **1** |  | |
| Yes | No | 21 273 | 2749 | | 12.9 | **2.14** | 2.06 | 2.23 | **1.71** | 1.64 | 1.78 |
| No | Yes | 99 506 | 21 946 | | 22.1 | **1** |  | | **1** |  | |
| Yes | Yes | 3421 | 1082 | | 31.6 | **1.64** | 1.52 | 1.77 | **1.35** | 1.25 | 1.46 |
| **Forceps delivery** | |  |  | |  |  |  |  |  |  |  |
| No | No | 1 135 180 | 74 516 | | 6.6 | **1** |  | | **1** |  | |
| Yes | No | 7137 | 710 | | 9.9 | **1.57** | 1.45 | 1.70 | **1.79** | 1.65 | 1.94 |
| No | Yes | 102 238 | 22 818 | | 22.3 | **1** |  | | **1** |  | |
| Yes | Yes | 689 | 210 | | 30.5 | **1.53** | 1.30 | 1.81 | **1.57** | 1.33 | 1.86 |
| **Retained placenta/membranes or invasive placenta** | | | | | | |  |  |  |  |  |
| No | No | 1 107 247 | 64 220 | | 5.80 | **1** |  | | **1** |  | |
| Yes | No | 35 070 | 11 006 | | 31.38 | **7.42** | 7.25 | 7.61 | **6.85** | 6.68 | 7.02 |
| No | Yes | 96 392 | 19 155 | | 19.9 | **1** |  | | **1** |  | |
| Yes | Yes | 6535 | 3873 | | 59.3 | **5.84** | 5.54 | 6.16 | **5.90** | 5.59 | 6.23 |
| **Obstetric trauma or laceration** | | | | | | | | | |  |  |
| No | No | 1 112 020 | 69 542 | | 6.25 | **1** |  | | **1** |  | |
| Yes | No | 30 297 | 5684 | | 18.76 | **3.46** | 3.35 | 3.56 | **2.48** | 2.40 | 2.56 |
| No | Yes | 97 441 | 21 010 | | 21.6 | **1** | Reference | | **1.0** |  | |
| Yes | Yes | 5486 | 2018 | | 36.8 | **2.12** | 2.00 | 2.25 | **1.74** | 1.63 | 1.84 |
| **Placental abruption** | |  |  | |  |  |  |  |  |  |  |
| No | No | 1 137 154 | 74 050 | | 6.51 | **1** | Reference | | **1** |  | |
| Yes | No | 5163 | 1176 | | 22.78 | **4.24** | 3.96 | 4.53 | **5.54** | 5.18 | 5.93 |
| No | Yes | 102 509 | 22 842 | | 22.3 | **1** | Reference | | **1** |  | |
| Yes | Yes | 418 | 186 | | 44.5 | **2.79** | 2.28 | 3.40 | **3.51** | 2.86 | 4.31 |
| **Placenta previa** | |  |  | |  |  |  |  |  |  |  |
| No | No | 1 140 458 | 74 860 | | 6.56 | **1** | Reference | | **1** |  | |
| Yes | No | 1859 | 366 | | 19.69 | **3.49** | 3.11 | 3.92 | **4.53** | 4.02 | 5.09 |
| No | Yes | 102 750 | 22 952 | | 22.3 | **1** | Reference | | **1** |  | |
| Yes | Yes | 177 | 76 | | 42.9 | **2.61** | 1.92 | 3.54 | **3.28** | 2.40 | 4.49 |
| **Dystocia** |  |  |  | |  |  |  |  |  |  |  |
| No | No | 1 110 430 | 70 772 | | 6.37 | **1** |  |  | **1** |  |  |
| Yes | No | 31 887 | 4454 | | 13.97 | **2.38** | 2.31 | 2.46 | **2.40** | 2.32 | 2.49 |
| No | Yes | 98 349 | 21 195 | | 21.6 | **1** |  | | **1** |  | |
| Yes | Yes | 4578 | 1833 | | 40.0 | **2.44** | 2.29 | 2.60 | **2.20** | 2.06 | 2.35 |
| **Live or perinatal death** | |  |  | |  |  |  |  |  |  |  |
| Live | No | 1 133 885 | 74 415 | | 6.56 | **1** |  | | **1** |  | |
| Stillborn | No | 5611 | 606 | | 10.80 | **1.73** | 1.58 | 1.88 | **2.13** | 1.96 | 2.33 |
| Early neonatal death | No | 2821 | 205 | | 7.27 | **1.02** | 0.97 | 1.07 | **1.55** | 1.35 | 1.80 |
| Live | Yes | 102 340 | 22 903 | | 22.38 | **1** |  | | **1** |  | |
| Stillborn | Yes | 435 | 91 | | 20.92 | **0.98** | 0.94 | 1.02 | **1.01** | 0.97 | 1.05 |
| Early neonatal death | Yes | 152 | 34 | | 22.37 | **1.01** | 0.94 | 1.08 | **1.06** | 0.99 | 1.13 |
| **Fetal sex in last pregnancy^c^** | |  |  | |  |  |  |  |  |  |  |
| Female | No | 555 917 | 37 675 | | 6.78 | **1** |  | | **1** |  | |
| Male | No | 586 371 | 37 545 | | 6.40 | **0.94** | 0.93 | 0.96 | **0.87** | 0.86 | 0.88 |
| Female | Yes | 49 962 | 11 420 | | 22.86 | **1** |  | | **1** |  | |
| Male | Yes | 52 964 | 11 608 | | 21.92 | **0.95** | 0.92 | 0.98 | **0.89** | 0.86 | 0.91 |

CI, confidence interval; OR, odds ratio; aOR, OR adjusted for marital status, period (1967– 1977, 1978–1987, 1988–1997, 1998–2007 and 2008–2017), maternal age, parity and WHO region of maternal birth

^a^ ICD-8 (international classification of diseases) codes 280–285; ICD-10 codes D50–D53, D55, D58–D61, D63 and D64

^b^ ICD-8 codes 286–289; ICD-10 codes D56, D57, D62, D65–D77, O460, O670 and O723

^c^ also adjusted for birthweight. Unspecified or unrecorded sex in 30 newborns
