# Supplementary material for: Recurrence of postpartum hemorrhage, maternal and paternal contribution, and the effect of offspring birthweight and sex: a population-based cohort study
Source: Arch Gynecol Obstet. 2022 Jan 9;306(5):1807–14. doi: 10.1007/s00404-021-06374-3 (PMC9519656; doi:10.1007/s00404-021-06374-3)
Supplement: Supplementary file 3 — Supplementary file3 Table S3: Inter-delivery interval and recurrence risk of postpartum hemorrhage (PPH, >500 ml). (DOCX 52 KB) [file 404_2021_6374_MOESM3_ESM.docx]

| **Supplementary Table 3. Inter-delivery interval and recurrence risk of postpartum hemorrhage (PPH, >500 ml)** | | | | | | | | | | |
| --- | --- | --- | --- | --- | --- | --- | --- | --- | --- | --- |
| **PPH in the previous delivery** | **PPH in the current delivery** | | | | | | | | |  |
|  | Interval (years) | Total | PPH (*n*) | % | **OR** | 95% CI | | **aOR** | 95% CI | |
| No | <1 | 5288 | 332 | 6.3 | **0.95** | 0.85 | 1.06 | **1.15** | 1.02 | 1.28 |
| No | 1 to <2 | 213 847 | 12 862 | 6.0 | **0.91** | 0.88 | 0.93 | **0.95** | 0.93 | 0.97 |
| No | 2 to <3 | 302 722 | 19 998 | 6.6 | **1** |  | | **1** |  | |
| No | 3 to <4 | 221 581 | 14 750 | 6.7 | **1.01** | 0.98 | 1.03 | **1.05** | 1.03 | 1.08 |
| No | 4 to <5 | 135 529 | 8752 | 6.5 | **0.98** | 0.95 | 1.00 | **1.06** | 1.03 | 1.09 |
| No | ≥5 | 263 350 | 18 532 | 7.0 | **1.07** | 1.05 | 1.09 | **1.12** | 1.10 | 1.15 |
| Yes | <1 | 365 | 86 | 23.6 | **1.05** | 0.83 | 1.34 | **1.10** | 0.85 | 1.41 |
| Yes | 1 to <2 | 20 522 | 4639 | 22.6 | **1.00** | 0.96 | 1.04 | **0.99** | 0.94 | 1.03 |
| Yes | 2 to <3 | 31 634 | 7165 | 22.6 | **1** |  | | **1** |  | |
| Yes | 3 to <4 | 20 916 | 4836 | 23.1 | **1.03** | 0.99 | 1.07 | **1.03** | 0.99 | 1.08 |
| Yes | 4 to <5 | 11 044 | 2444 | 22.1 | **0.97** | 0.92 | 1.02 | **0.97** | 0.92 | 1.02 |
| Yes | ≥5 | 18 446 | 3858 | 20.9 | **0.90** | 0.86 | 0.94 | **0.86** | 0.82 | 0.90 |

aOR, OR adjusted for marital status, period (1967–1977, 1978–1987, 1988–1997, 1998–2007 and 2008–2017), maternal age, parity and WHO region of maternal birth
