# Supplementary figures and images for: Recurrence of postpartum hemorrhage, maternal and paternal contribution, and the effect of offspring birthweight and sex: a population-based cohort study
Source: Arch Gynecol Obstet. 2022 Jan 9;306(5):1807–14. doi: 10.1007/s00404-021-06374-3 (PMC9519656; doi:10.1007/s00404-021-06374-3)

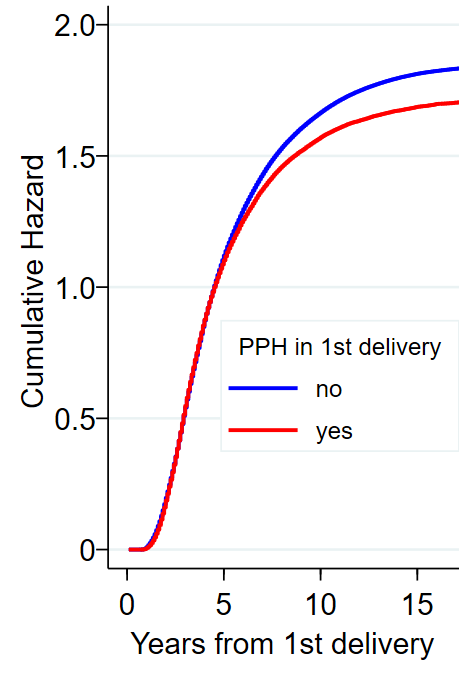

Supplement: Supplementary file 4 — Supplementary file4 Figure S1: Cumulative Hazards of the second delivery according to postpartum hemorrhage in the first delivery, adjusted for period and maternal age. (TIF 92 KB) [file 404_2021_6374_MOESM4_ESM.tif]

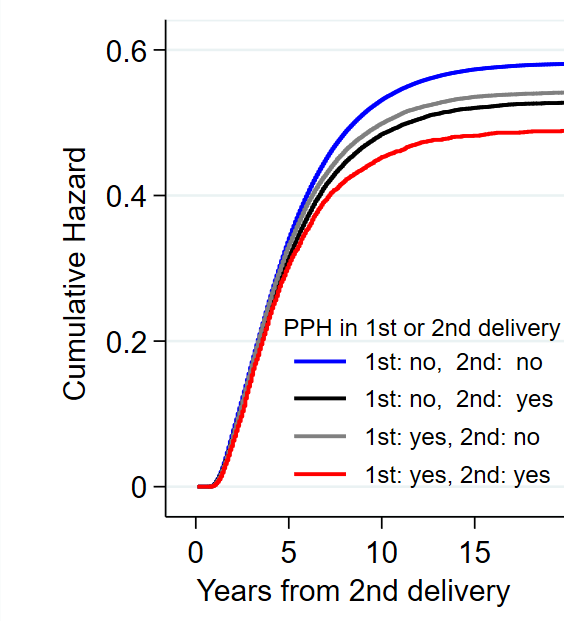

Supplement: Supplementary file 5 — Supplementary file5 Figure S2: Cumulative Hazards of the third delivery according to postpartum hemorrhage in the first or second delivery, adjusted for period and maternal age. (TIF 123 KB) [file 404_2021_6374_MOESM5_ESM.tif]
