# Supplementary material for: Recurrence of postpartum hemorrhage, maternal and paternal contribution, and the effect of offspring birthweight and sex: a population-based cohort study
Source: Arch Gynecol Obstet. 2022 Jan 9;306(5):1807–14. doi: 10.1007/s00404-021-06374-3 (PMC9519656; doi:10.1007/s00404-021-06374-3)
Supplement: Supplementary file 6 — Supplementary file6 Statistical analysis. (DOCX 39 KB) [file 404_2021_6374_MOESM6_ESM.docx]

### Supporting information Statistical analysis

### Independent variables

The main independent variables were a history of PPH (postpartum hemorrhage) in a previous delivery and the birthweight in the current delivery. To assess temporal changes in the occurrence of PPH, we divided the population into birth-year periods. Further, we investigated whether the occurrence and recurrence of PPH were influenced by maternal conditions such as pregestational and gestational diabetes mellitus, chronic hypertension, preeclampsia, operative vaginal delivery (forceps or vacuum), shoulder dystocia and uterine atony. The possible effect of fetal sex on the recurrence risk of PPH was also explored.

These analyses included the following possible confounding factors: maternal age (<20 years, 20–24 years, 25–29 years, 30–34 years, 35–39 years or ≥40 years), parity (0, 1, 2, 3 or ≥4 ), inter-delivery interval (<1 year, 1 to <2 years, 2 to <3 years, 3 to <4 years, 4 to <5 years or ≥5 years), marital status (married/registered partner, cohabitating, not married/alone, divorced/separated/widow, not defined), mother’s country of birth (Norway or eight WHO regions)([1](#_ENREF_1)) [(A) high-income countries, (B) Central Europe, Eastern Europe and Central Asia, (C) sub-Saharan Africa, (D) North Africa and Middle East, (E) South Asia, (F) Southeast Asia, East Asia and Oceania, (G) Latin America and Caribbean or (H) unknown or stateless] and level of education (available until 2013) (<8 years, 8–10 years, 11–12 years, 13–17 years, ≥18 years or no information). When analyzing recurrence, the period of birth was divided into five groups with approximately equal durations (1967–1977, 1978–1987, 1988–1997, 1998–2007 and 2008–2017).

## Statistical analysis

We used logistic regression analyses to calculate odds ratios (ORs) with 95% confidence intervals (CIs) for PPH in the actual birth as the outcome, and a history of previous PPH as the main exposure variable. We accounted for the hierarchical nature of the family data by performing multilevel regression analyses in which the data were divided into different levels—in analyses including pairs of births of the same parent: current delivery (level 1) and parent (level 2). Possible confounding variables were included if they were associated with PPH in both the current and previous births of the same parent.

We used sensitivity analyses to assess the impact of unmeasured confounders on the recurrence of PPH between deliveries.([2](#_ENREF_2)) We performed a Markov Chain Monte Carlo simulation([3](#_ENREF_3)) with the prior assumption that adding an influential, unmeasured confounder to known confounder(s) would zero out the recurrence risk, which decreased the regression coefficient (β; standard deviation) for the main exposure variable of PPH to 0; 0.05, corresponding to an OR of 1 with a 95% CI of 0.9 to 1.1.

To estimate the proportion of all cases of PPH attributable to previous PPH and any category of birthweight ≥4000 g in the current delivery, adjusted population attributable fractions (aPAFs) were calculated:([4](#_ENREF_4))

aPAF= $pd\frac{aRR-1}{aRR}$ and 1– $\sum_{i=0}^{k} \frac{pd_{i}}{{aRR}_{i}}$

for two or more exposure categories, respectively, where *pd_i_* is the proportion of PPH cases in the *i*^th^ exposure category among all cases, and *aRR_i_* is the adjusted relative risk in the *i*^th^ exposure category compared with the unexposed group (reference, *i*=0). We calculated aPAF for PPH in the same mother with a history of PPH or birthweight (<4000 g, 4000–4499 g, 4500–4999 g and ≥5000 g) in the current delivery as the exposure variable.

To assess likelihoods of a further delivery after PPH, we calculated further pregnancy rate, defined as the percentage of women who had å further delivery after the first,([5](#_ENREF_5)) and used Cox proportional hazards regression of time to a subsequent delivery, adjusting for possible confounding factors in the previous delivery. Logistic regression, adjusting for period of delivery, revealed no significant differences between women with and without PPH in maternal death (0.4 and 0.5%, respectively) or emigration (0.5 and 0.6%). Data on women who did not have a subsequent delivery were censored observations, with censored time equal to the last date of registration (31 December 2017).

The statistical analyses were performed using SPSS (version 25) and MLwiN (version 3.05).

**References**

1. World Health Organization. Office of World Health Reporting. (2002). The World health report: 2002: reducing risks, promoting healthy life: overview. World Health Organization. [Available from: <https://www.who.int/whr/2002/en/>].

2. Greenland S. Bayesian perspectives for epidemiologic research: III. Bias analysis via missing-data methods. Int J Epidemiol. 2009;38(6):1662-73.

3. Rasbash JS, F.; Browne, W.J.; Goldstein, H. A User’s Guide to MLwiN, v3.03. Centre for Multilevel Modelling. 2019.

4. Rockhill B, Newman B, Weinberg C. Use and misuse of population attributable fractions. Am J Public Health. 1998;88(1):15-9.

5. Rasmussen S, Irgens LM, Dalaker K. The effect on the likelihood of further pregnancy of placental abruption and the rate of its recurrence. British journal of obstetrics and gynaecology. 1997;104(11):1292-5.
